# Reconstruction of gene regulatory networks for *Caenorhabditis elegans* using tree-shaped gene expression data

**DOI:** 10.1093/bib/bbae396

**Published:** 2024-08-12

**Authors:** Yida Wu, Da Zhou, Jie Hu

**Affiliations:** School of Mathematical Sciences, Xiamen University, Zengcuo'an West Road, Siming District, Xiamen 361000, China; School of Mathematical Sciences, Xiamen University, Zengcuo'an West Road, Siming District, Xiamen 361000, China; School of Mathematical Sciences, Xiamen University, Zengcuo'an West Road, Siming District, Xiamen 361000, China

**Keywords:** gene regulatory network, tree-shaped gene expression data, data integration, Boolean network, Bayesian statistics

## Abstract

Constructing gene regulatory networks is a widely adopted approach for investigating gene regulation, offering diverse applications in biology and medicine. A great deal of research focuses on using time series data or single-cell RNA-sequencing data to infer gene regulatory networks. However, such gene expression data lack either cellular or temporal information. Fortunately, the advent of time-lapse confocal laser microscopy enables biologists to obtain tree-shaped gene expression data of *Caenorhabditis elegans*, achieving both cellular and temporal resolution. Although such tree-shaped data provide abundant knowledge, they pose challenges like non-pairwise time series, laying the inaccuracy of downstream analysis. To address this issue, a comprehensive framework for data integration and a novel Bayesian approach based on Boolean network with time delay are proposed. The pre-screening process and Markov Chain Monte Carlo algorithm are applied to obtain the parameter estimates. Simulation studies show that our method outperforms existing Boolean network inference algorithms. Leveraging the proposed approach, gene regulatory networks for five subtrees are reconstructed based on the real tree-shaped datatsets of *Caenorhabditis elegans*, where some gene regulatory relationships confirmed in previous genetic studies are recovered. Also, heterogeneity of regulatory relationships in different cell lineage subtrees is detected. Furthermore, the exploration of potential gene regulatory relationships that bear importance in human diseases is undertaken. All source code is available at the GitHub repository https://github.com/edawu11/BBTD.git.

## Introduction

Gene regulatory network (GRN) is a collection of molecular species and their interactions, together controlling gene-product (RNA and proteins) abundance [1]. Constructing GRNs enables biologists to shed light on the biological processes of an organism from a holistic perspective [2, 3]. There are two main types of gene expression data used for GRN construction. First, time series data, produced by DNA microarray or next-generation RNA-sequencing, provide an opportunity for scientists to investigate GRN by leveraging the temporal pattern [4]. However, such data obscure biological signals in the gene expression profiles on cellular levels, which is insufficient to explore gene regulation for specific cell types [5]. Second, single-cell RNA-sequencing data offer a profound insight into GRNs at cellular levels, but these data lack temporal information of genes, which is crucial to reflect the genes’ dynamic change within cells [5].

**Figure 1:**
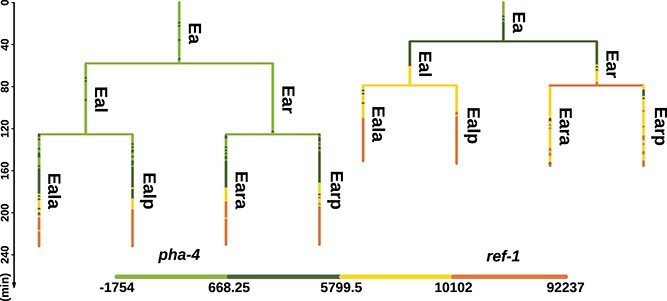
Comparison of two cell lineage subtrees; the figure shows a part of two cell lineage subtrees, which are, respectively, derived from two real data files: *CD20060629_pha4_b2.csv* and *CD20081014_ref-1_2_L1.csv*; both subtrees start from ‘Ea’ cell at time zero; the length of each vertical line corresponds to the lifetime of a single cell; each horizontal line represents an event of cell division; the color of lines corresponds to the fluorescent intensity of two labeled gene: *pha-4* and *ref-1*; the cell names are from [9], which are signed on the right side of each vertical line.

With the development of modern high throughput experiment techniques, there is a chance to construct GRNs by leveraging the gene expression data including both cellular and temporal information. [6] and [7] described an automated system for analyzing successive gene expression profiles in *Caenorhabditis elegans* (*C. elegans*) with cellular resolution from the zygote until adult using time-lapse confocal laser microscopy. Specifically, a *C. elegans* individual is measured once per 1.5 min to concurrently report the fluorescence intensity of labeled gene within each living cell. Thus, this technology enables the generation of temporal data for each cell within a single worm, tracking from its birth through to division or death, all recorded within a single data file. Consequently, each data file documents the quantified fluorescent intensity of one specific gene in a single *C. elegans* individual and different data files correspond to different worms, which are provided by [8]. Also, each data file can be depicted as a binary tree, as shown in Fig. 1, clearly displaying parent–child cell relationships. Hence, we refer to this kind of cellular-temporal data as tree-shaped gene expression data. Furthermore, due to the invariant cell lineage from zygote to adult of *C. elegans* [9], which means all *C. elegans* individuals follow exactly the same path to develop from embryo into adult worm, we can summarize each data file into specific cell lineage subtrees. Following the characterization by [9], there are mainly five distinct cell lineage subtrees of *C. elegans*, which include all descendants of respective founder cells: ‘AB’, ‘C’, ‘D’, ‘E’, and ‘MS’. These five subtrees are denoted with the same names as their founder cells. Due to the absence of differentiation in the early stages and the limited duration of observation, only a small fraction of the descendants in each subtree manifest cell fates. However, the cell fates across different subtrees are markedly distinct. For example, the ‘AB’ subtree predominantly differentiates into hypodermis and neuronal cells, while the ‘E’ subtree mainly gives rise to intestinal cells. Therefore, it is essential to reconstruct the GRN for each subtree independently, as similarly demonstrated in [10].

Although such tree-shaped data provide valuable insights about cell lineage, they pose a challenge to downstream analysis. Due to the limitation of tracking only one labeled gene per individual, the lifetimes of the same cell in different data files may not align [8]. For instance, as shown in Fig. 1, the lifetime of ‘Eal’ within the left subtree is longer than the right one, resulting in non-pairwise data points measured as the measurement intervals are consistently 1.5 min. Thus, it is impractical to directly align the fluorescence intensity from different data files according to the absolute time. To solve the problem of non-pairwise time series, [10] resorted to averaging the fluorescence intensity of each cell to one averaged value which can be applied to perform downstream analysis. However, since the fluorescent intensity may vary greatly within a cell, this method would result in the loss of temporal information at cellular levels. In order to avoid this drawback, a framework of data integration including interpolation and binarization is proposed, which enables each cell from different data files to have identical and matched time points.

With the availability of cellular and temporal information, several methods of GRN reconstruction for *C. elegans* were proposed previously. [11] developed a mathematical model on wild-type data to predict gene regulatory relationships for *C. elegans*, but only focused on a very small number of ‘C’ cells. [10] employed a probabilistic graphical model to reconstruct GRNs. While their analysis centered around five founder cells and their descendants, it is noteworthy that their approach was dependent on known protein–protein interaction, protein–DNA interaction, and gene knockout data. Hence, there is no research of GRN reconstruction focusing on distinct cell lineage subtrees for *C. elegans* by leveraging tree-shaped data exclusively. Due to the appealing characteristics of dynamic complexity and robustness to noisy data [2, 12, 13], employing Boolean networks for reconstruction of GRNs is a proper strategy to handle such high-resolution but rather noisy datasets. REVEAL (REV) [14] and Best-Fit Extension (BFE) [15] are two widely recognized algorithms for inferring Boolean networks. Despite that, the former method primarily employs a deterministic Boolean network model, which has limited capacity to accommodate biological uncertainties, and the latter focuses on local optimization, rather than optimizing the entire network jointly. Recently, ATEN [16] has been developed to infer Boolean network topology and dynamics from noisy time series data. However, its reliance on deterministic Boolean networks limits its robustness to uncertainty. [17] proposed a probabilistic Boolean network on the protein interaction network of yeast cell cycle. This stochastic model, which is stable and robust when considering a wide range of noise, can be used as a tool to study interactions among genes [18–22]. However, this work has some certain limitations. First, gene regulation was modeled by fixing the network’s time delay as one time unit, thereby oversimplifying the underlying dynamics of gene regulation [23, 24]. Second, they did not include the development of a parameter inference algorithm for the model. Therefore, it is necessary to address the problem within the stochastic model proposed by [17] and develop a new method to reconstruct GRNs.

In the following contents, a framework of data integration is proposed to transform tree-shaped data with non-pairwise time series into pairwise data for five distinct cell lineage subtrees. Next, a novel **B**ayesian approach based on **B**oolean network with **t**ime **d**elay (BBTD) is developed. Especially, time delay is introduced as a discrete parameter in our model, allowing for a more realistic representation of gene regulatory processes [25–27]. Finally, BBTD is applied to reconstruct GRNs for each subtree based on both synthesized and real data.

## Materials and methods

### Data integration

**Figure 2:**
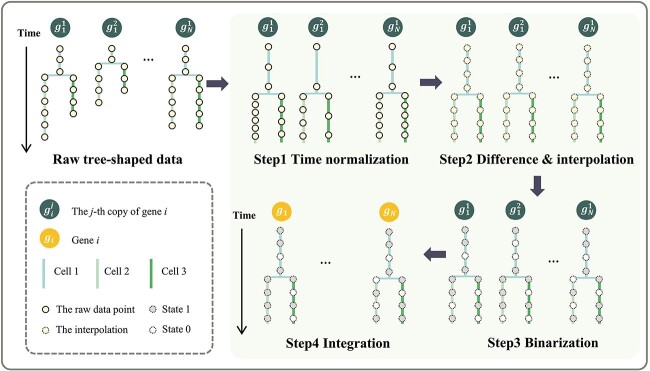
A four-step process of data integration for each cell lineage subtree; Step 1: normalize the lifetime of each cell to a standardized unit; Step 2: compute the first-order difference of the raw data and interpolate them for each cell to the same number; Step 3: discretize the expression rates into binary values; Step 4: merge the multiple copies of the same gene.

The tree-shaped gene expression dataset for *C. elegans* provided by [8] is downloaded from http://epic.gs.washington.edu/. There are 184 files within the dataset, corresponding to 105 genes, and the repeated files of one specific gene are called copies. In order to reduce impacts of noise fluctuation, only the gene expression onset cells, which are provided by [28], with their descendants are considered in each file for data integration. Besides, since the fluorescent intensity associated with gene expression tends to remain stable or increase over time, rarely exhibiting a decrease [28], which can not reflect the real expression state of change, the first-order difference of fluorescence intensity is computed, defined as expression rate. As mentioned in Section 2, this tree-shaped dataset presents a primary challenge for application since the lifetimes of one cell may vary across embryos. Hence, it is not feasible to directly align the time series data from different files based on the absolute time. To address this, as depicted in Fig. 2, a data integration framework to align time series is proposed for each cell lineage subtree as follows:

- Step 1: normalize the lifetime of each cell to a standardized unit.
- Step 2: compute expression rates and interpolate the same number of points to each cell using FMM spline method [29]. The number of interpolations for each subtree is the median of the number of points per cell, which are shown in Table 1. For convenience, all data points are referred to as interpolations after Step 2.
- Step 3: discretize the expression rates into binary values, where $1$ indicates high expression rate and $0$ denotes low expression rate. The threshold is set as the median values of expression rates within each subtree.
- Step 4: merge the multiple copies of the same gene. At each gene expression time point, if more than half of the gene copies exhibit State $1$, the gene state is classified as $1$. Conversely, the gene state is categorized as $0$. If all copies of one gene are missing at some gene expression time points, then the gene states at that time are set to missing values.

**Table 1:** Summary of five cell lineage subtree datasets; the ‘Gene’ column shows the number of candidate genes and the ‘Cell’ column shows the number of candidate cells; the ‘Inters’ column displays the number of interpolations per cell for analysis.

Subtree	Gene	Cell	Inters
AB	27	112	24
C	30	51	20
D	20	12	28
E	40	26	16
MS	29	80	22

After the integration of raw tree-shaped data for each subtree, it is observed that missing values persist within each subtree dataset (see Supplementary Figure S1). To alleviate the impacts of missing values, candidate cells and genes are selected for the subsequent analysis, which are displayed in Supplementary Table S1 and Table S2. Then, the remaining missing values ($<5\%$ in each subtree, see Supplementary Figure S2) are treated as low expression rates, i.e. State 0. The details of selection are shown in Supplementary I. Table 1 provides a summary of five subtree datasets used for network inference.

### BBTD method

To effectively capture the dynamic nature of gene regulation from pairwise time series subtree dataset, a new method, i.e. BBTD, is proposed. This method comprises a probabilistic Boolean network model with time delay, a pre-screening process, and a Bayesian inference framework. The details of BBTD are discussed below.

#### Statistical modeling

**Figure 3:**
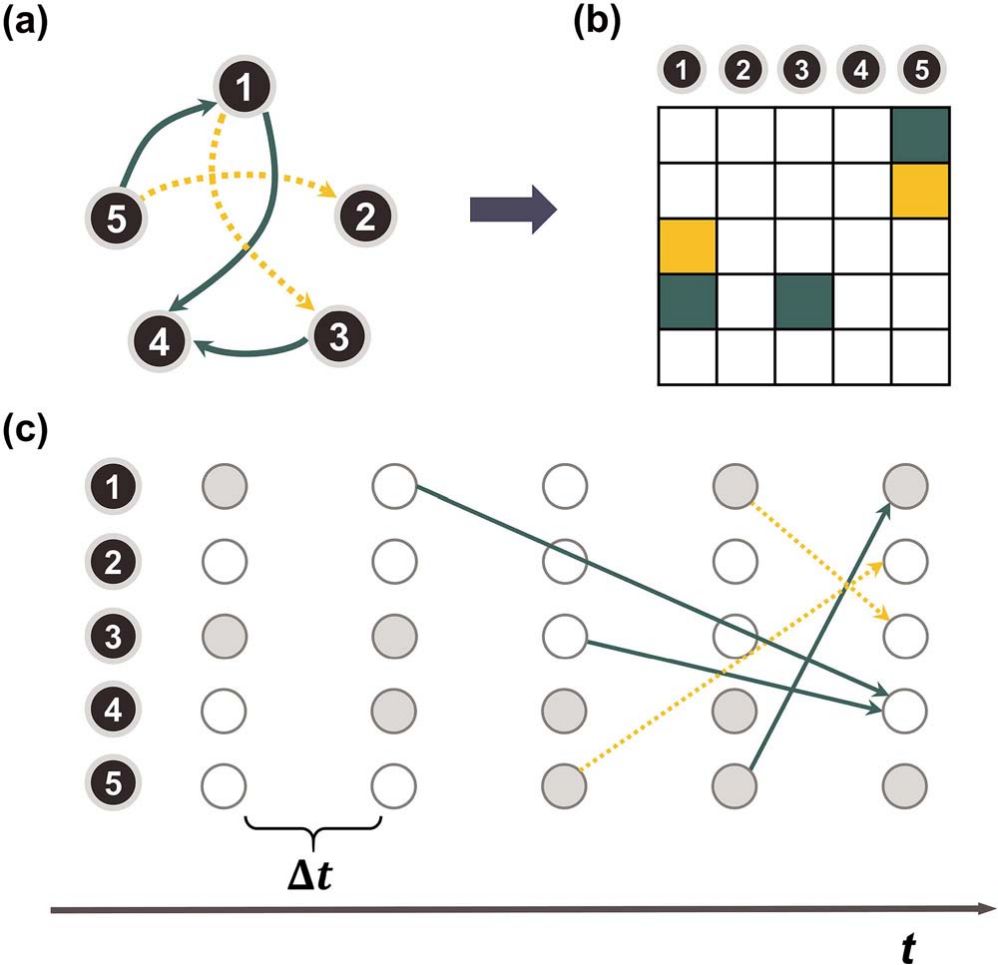
An example of the probabilistic Boolean network model with time delay; (a) depicts a 5-gene GRN; each node corresponds to a gene, and the regulatory relationships are illustrated by solid deep green arrows (indicating positive regulations) and dashed orange arrows (indicating negative regulations); (b) presents the matrix $\mathbf{A}$ corresponding to the GRN representation in (a); a deep green grid signifies $a_{ij}=1$, an orange grid represents $a_{ij}=-1$, and a white grid represents $a_{ij}=0$; (c) demonstrates the state transition process of the 5-gene GRN; the gray circle represents an active state of the gene, while the white circle represents an inactive state; specifically, (c) illustrates the regulatory mechanism governing the gene states at time $t$ with different time delay as an example.

To begin with, we consider a network evolving in the configuration space $S=\{0,1\}^{N}$ with $N$ being the number of genes, where $1$ denotes active state (high expression rate) and $0$ indicates inactive state (low expression rate). Then, an $N$-dimensional square matrix $\mathbf{A} = \left (a_{i j}\right ) (i,j=1,\ldots , N)$ is utilized to represent the GRN which includes $N$ genes, where $a_{ij} \in \{1,-1,0\}$. Specifically, $a_{ij}=1$ means a positive regulation acting from regulator gene $j$ to regulated gene $i$, which indicates that when gene $j$ reaches an active state, gene $i$ will receive a positive signal and is more likely to be activated after some time. $a_{ij}=-1$ means when gene $j$ reaches an active state, gene $i$ will receive a negative sign and is more likely to be suppressed after some time. $a_{ij}=0$ indicates that there is no regulation emitted from gene $j$ to gene $i$. Since the binarization ignores the continuous and often subtle variations in gene expression levels that are biologically important, especially in the context of self-regulation where feedback mechanisms can be sensitive to small changes in expression levels [30], self-regulation of genes is not considered in our study. Hence, as in [31–33], we do not consider self-regulation in this case and set $a_{ii}=0, \forall i=1,\ldots ,N$. Figure 3a provides an illustrative example of five-gene GRN. According to the definition of matrix $\mathbf{A}$ above, the GRN can be represented by a five-dimensional square matrix as shown in Fig. 3b.

Furthermore, it is crucial to account for time delay between the regulator gene and the regulated gene in our analysis. To address this problem, a time delay matrix is denoted as $\boldsymbol{\Delta } = \left (\delta _{ij}\right ), i=1,\ldots N$. When $a_{ij}\neq 0$, $\delta _{ij} \in \{0,\Delta t,2\Delta t,3\Delta t,4\Delta t,5\Delta t\}$ represents the time delay when gene $j$ activates or suppresses gene $i$, where $\Delta t$ denotes the time unit between two successive interpolations. This is exemplified in Fig. 3c, where gene 1 suppresses gene 3 with one unit of time delay, while gene 3 activates gene 4 with two units of time delay. Thus, the joint probability of state transition is defined as follows: 


(1) \begin{align*}& \begin{split} &\mathrm{P}\left(s_{1}^{t^{r}_{k}}, \ldots, s_{N}^{t^{r}_{k}} \mid s_{1}^{t^{r}_{k}-\delta_{11}}, \ldots, s_{N}^{t^{r}_{k}-\delta_{1N}},\ldots,s_{1}^{t^{r}_{k}-\delta_{N1}},\ldots,s_{N}^{t^{r}_{k}-\delta_{NN}}\right) \\ & \quad = \prod_{i=1}^{N} \mathrm{P}\left(s_{i}^{t^{r}_{k}} \mid s_{1}^{t^{r}_{k}-\delta_{i1}}, \ldots, s_{N}^{t^{r}_{k}-\delta_{iN}}\right), \end{split}\end{align*}

where $s_{i}^{t^{r}_{k}} \in \{0,1\}$ denotes the expression state of gene $i (i=1,\ldots ,N)$ at time $t^{r}_{k} (r = 1,\ldots ,R,k =6,\ldots ,K^{r})$. $R$ denotes the number of root cells, i.e. cells that do not have a parent among the candidate cells. $K^{r}$ indicates the total number of interpolations for the $r$-th root cell as well as its daughter cells. Since the max time delay as defined is $5 \Delta t$, $t^{r}_{k}$ begins at $k=6$ for $\forall r=1,\ldots ,R$. The equality in Equation (1) is true for the reason of conditional independence, that is, when given all of the regulator genes’ states of the regulated gene $i$, the state of gene $i$ is independent of those of the other genes. Then, we denote $H_{i}^{t^{r}_{k}}=\sum _{j=1}^{N} a_{i j} s_{j}^{{t^{r}_{k}}-\delta _{ij}}$ being the input of all signals received of gene $i$ at time $t_{k}^{r}$ and the conditional probability of $s_{i}^{t_{k}^{r}}$ is given as follows: If $H_{i}^{t^{r}_{k}}= 0$, 


(2) \begin{align*}& \begin{aligned}[b] \mathrm{P}\left(s_{i}^{t^{r}_{k}}=s_{i}^{t^{r}_{k}-\Delta t} \mid s_{i}^{t^{r}_{k}-\Delta t} \right)=\frac{1}{1+e^{-\alpha}}; \end{aligned}\end{align*}

and $H_{i}^{t^{r}_{k}} \neq 0$, 


(3) \begin{align*} \mathrm{P}\left(s_{i}^{t^{r}_{k}}\mid s_{1}^{t^{r}_{k}-\delta_{i1}}, \ldots, s_{N}^{t^{r}_{k}-\delta_{iN}}\right)\ =&\ \frac{\exp \left[ \beta (2s_{i}^{t^{r}_{k}}-1)H_{i}^{t^{r}_{k}}\right]}{\exp (\beta H_{i}^{t^{r}_{k}})+\exp (-\beta H_{i}^{t^{r}_{k}})}.\end{align*}

Note that the positive parameter $\alpha $ in Equation (2) controls the probability for gene $i$ to maintain its state when the input to gene $i$ is zero [17]. And the positive temperature-like parameter $\beta $ in Equation (3) represents noise in the system from the perspective of statistical physics [17, 34, 35].

#### Pre-screening process

In the proposed Boolean network model, the unknown parameters of $\mathbf{A}=(a_{ij})$ is $N(N-1)$. As $N$ increases, the number of $a_{ij}$ to be estimated increases at a polynomial level. To improve the efficiency of network inference, we design a pre-screening process based on Fisher’s exact test [36] to reduce the number of unknown parameters.

Fisher’s exact test is a hypothesis test for testing significant differences in contingency tables. As introduced in Section 2, expression state $s_{i}^{t}$ is a binary variable. Let $\boldsymbol{s}_{i}^{\boldsymbol{t}-m\Delta t}=\left (s_{i}^{t^{1}_{6}-m\Delta t},\ldots ,s_{i}^{t^{R}_{K^{R}}-m\Delta t}\right )$ be the vector of all available expression states of gene $i$, where $m=0,\ldots ,5$. Then, a contingency table is created to summarize the expression states of a regulated gene and a candidate regulator gene. Assuming that if the *P* -value of Fisher’s exact test is greater than or equal to a given threshold value $\theta $, there is no existing regulatory relationship between these two genes. The scheme of the pre-screening process is displayed in Algorithm 1. After that, the $a_{ij}$ whose estimated value is $\tilde{a}_{ij}=0$ no longer participates in subsequent calculations, thereby reducing the number of estimated parameters.


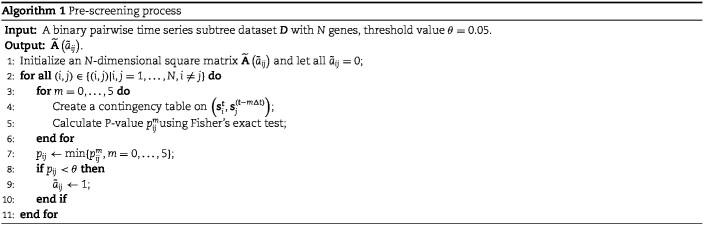


#### Bayesian inference

After implementing the pre-screening process, the unknown parameters are estimated under the Bayesian framework. In order to obtain the posterior of these parameters, the priors need to be specified. Initially, it is assumed that the regulated cases of each gene are independent of each other, and that the corresponding time delay is meaningful only if the regulatory relationship exists. Thus, the joint prior $\pi \left (\mathbf{A},\mathbf{\Delta } \right )$ is given as follows: 


(4) \begin{align*}& \begin{aligned}[b] \pi\left(\mathbf{A},\boldsymbol{\Delta}\right) &= \pi\left(\mathbf{A}\right) \pi\left(\boldsymbol{\Delta} \mid \mathbf{A} \right)\\ &= \prod_{i=1}^{N} \pi\left(a_{i.}\right) \prod_{\substack{j=1\\j\neq i}}^{N} \pi\left(\delta_{ij} \mid a_{ij}\right), \end{aligned}\end{align*}

where 


(5) \begin{align*} \mathrm{\pi}\left(a_{i.}\right)&=\mathbf{I}_{\{x_{i}=0\}}\kappa+\mathbf{I}_{\{x_{i}\neq0\}}(1-\kappa)c(\lambda)\frac{\exp(-\lambda x_{i})}{\binom{N-1}{x_{i}}}\frac{1}{2^{x_{i}}}, \end{align*}

(6) \begin{align*} \pi\left(\delta_{ij} \mid a_{ij}\right)&=\mathbf{I}_{\{a_{ij} \neq 0\}} \pi_{1}\left(\delta_{ij}\right) + \mathbf{I}_{\{a_{ij} = 0\}}\pi_{2}\left(\delta_{ij}\right).\end{align*}

In Equation (5), $a_{i.}$ indicates the $i$-th row of $\mathbf{A}$, and $x_{i} = \sum _{\substack{j=1\\j\neq i}}\mathbf{I}_{\{a_{ij} \neq 0 \}}$ means the number of candidate regulatory relationships whose regulated gene is gene $i$. According to [37], GRN is considered sparse. Then, two hyperparameters: $\lambda (\lambda>0)$ and $\kappa (0<\kappa <1)$ are introduced to control the sparsity. $c(\lambda )$ is the normalizing constant of $\exp{(-\lambda{x_{i}})}$. In Equation (6), when $a_{ij} \neq 0$, $\delta _{ij}$ is assigned a non-informative prior, i.e. uniform discrete distribution, defined as 


(7) \begin{align*}& \begin{aligned}[b] \pi_{1}\left(\delta_{ij}\right) &= \left\{\begin{array}{@{}lr} \frac{1}{6}, & \delta_{ij} \in \{0,\Delta t,2\Delta t,3\Delta t,4\Delta t,5\Delta t\} \\ 0, & \text{otherwise} \end{array}\right.\!. \end{aligned}\end{align*}

When $a_{ij}=0$, the corresponding $\delta _{ij}$ is redundant and should be fixed to $0$. However, fixing $\delta _{ij}$ leads to a changing dimension problem, rendering the sampling algorithm unaccessible. To address this, a spike-and-slab prior is implemented for $\delta _{ij}$ when $a_{ij}=0$, ensuring consistent dimensionality throughout iterations [38]. This prior is given as follows: 


(8) \begin{align*}& \begin{aligned}[b] \pi_{2}\left(\delta_{ij}\right) &= \left\{\begin{array}{@{}lr} 0.999, & \delta_{ij} = 0 \\ 0.0002, & \delta_{ij} \in \{\Delta t,2\Delta t,3\Delta t,4\Delta t,5\Delta t\} \\ 0, & \text{otherwise} \end{array}\right.. \end{aligned}\end{align*}

Then, for two positive parameters $\alpha $ and $\beta $ in the model as well as two hyperparameters $\lambda $ and $\kappa $, their priors are supposed as follows: 


\begin{align*} \begin{split} &\alpha \sim \Gamma\left(1,10\right),\\ &\beta \sim \Gamma\left(100,100\right),\\ &\lambda \sim \Gamma\left(490,70\right),\\ &\kappa \sim \text{Beta}\left(6,24\right). \end{split}\nonumber\end{align*}

Finally, the full joint posterior can be written as follows: 


(9) \begin{align*} \begin{split} & \pi\left(\mathbf{A}, \boldsymbol{\Delta},\alpha,\beta, \lambda,\kappa \mid \boldsymbol{D}\right) \\ = &\prod_{r=1}^{R}\prod_{k=6}^{K^{r}}\prod_{i=1}^{N} \mathrm{P}\left(s_{i}^{t^{r}_{k}} \mid s_{1}^{t^{r}_{k}-\delta_{i1}}, \ldots, s_{N}^{t^{r}_{k}-\delta_{iN}}\right) \cdot \\&\pi \left(\mathbf{A},\boldsymbol{\Delta} \right) \pi(\alpha)\pi(\beta)\pi(\lambda)\pi(\kappa), \end{split}\end{align*}

where $\boldsymbol{D}$ represents the binary pairwise time series subtree dataset.

After getting the posterior, Markov Chain Monte Carlo (MCMC) is performed to draw samples from Equation (9). Specifically, Gibbs Sampling [39] and Metropolis–Hastings (M-H) algorithm [40, 41] are utilized to draw samples of unknown parameters. For discrete parameters $\left (\mathbf{A},\boldsymbol{\Delta }\right )$, their samples are drawn by Gibbs Sampling. The key step for Gibbs Sampling is to obtain the conditional posterior of the parameters. For each $\left (a_{ij},\delta _{ij}\right )$, their conditional posterior is given as follows: 


(10) \begin{align*} \begin{split} &\pi\left(a_{ij}, \delta_{ij} \mid -\right) \\ \propto&\prod_{r=1}^{R}\prod_{k=6}^{K^{r}}\mathrm{P}\left(s_{i}^{t^{r}_{k}} \mid s_{1}^{t^{r}_{k}-\delta_{i1}}, \ldots, s_{N}^{t^{r}_{k}-\delta_{iN}}\right) \pi\left(a_{i.}\right)\pi\left(\delta_{ij} \mid a_{ij}\right), \end{split}\end{align*}

where ‘−’ means given all other variables. For continuous parameters $\alpha $, $\beta $, $\lambda $, and $\kappa $, the M-H algorithm is used to draw samples. The complete scheme of MCMC algorithm is shown in Algorithm 2. When sampling is completed, a burn-in phase is conducted by discarding the first half of the samples to ensure convergence to the posterior. Finally, the Maximum-A-Posteriori estimate $\left (\widehat{\mathbf{A}}, \widehat{\boldsymbol{\Delta }},\hat{\alpha },\hat{\beta },\hat{\lambda },\hat{\kappa }\right )$ is calculated based on the remaining samples.


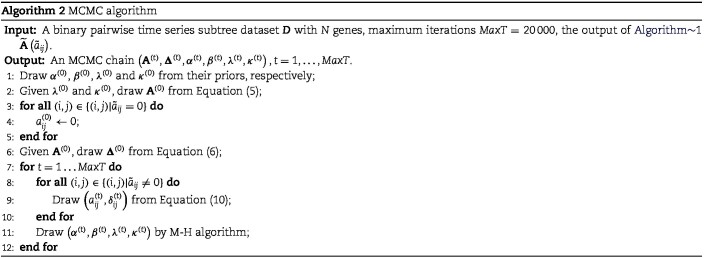


## Results

### Simulation studies

The synthesized tree-shaped datasets are generated from the probabilistic Boolean network model in Equations (2) to (3) the number of whose genes and cells, and the number of interpolations per cell are consistent with the real datasets. The ground truth values of parameters are drawn from their priors. For each subtree, 50 groups of synthesized datasets are generated and BBTD is utilized to infer their gene regulatory relationships.

To begin with, the pre-screening process (Algorithm 1) is applied to filter the discrete parameters $\mathbf{A}=\left (a_{ij}\right )$. After the pre-screening process is completed, two indices are introduced to evaluate the performance of the pre-screening process, which are defined as follows:

- Pre-screening Rate (PR) of $\widetilde{\mathbf{A}}$,
  (11) \begin{align*}& \begin{split} \text{PR}=\frac{\#\{\tilde{a}_{ij}=0\}}{\#\{\tilde{a}_{ij}\}}.\qquad\qquad\qquad\qquad \end{split}\end{align*}

- False Negative Rate (FNR) of $\widetilde{\mathbf{A}}$,
  (12) \begin{align*}& \begin{split} \text{FNR}=\frac{\sum_{i\neq j}{\mathbf{I}_{\{\tilde{a}_{ij} = 0 \& a_{ij} \neq 0\}}}}{\sum_{i\neq j}\mathbf{I}_{\{a_{ij} \neq 0\}}}.\qquad\qquad\qquad \end{split}\end{align*}

Next, MCMC algorithm (Algorithm 2) is performed with three independent MCMC chains for each group. Since the discrete parameters $\mathbf{A}=\left (a_{ij}\right )$ where we are interested are sparse, i.e. there are excessive zeros in $\mathbf{A}$ and $\widehat{\mathbf{A}}$, five indices are used to evaluate the performance of simulation that are defined as bellow:

- True Positive Rate (TPR) of $\widehat{\mathbf{A}}$,
  (13) \begin{align*}& \begin{split} \text{TPR}=\frac{\sum_{i\neq j}{\mathbf{I}_{\{\hat{a}_{ij} = 1 \& a_{ij} = 1\}}}}{\sum_{i\neq j}{\mathbf{I}_{\{a_{ij} = 1\}}}}.\qquad\qquad\qquad \end{split}\end{align*}

- Precise Positive Rate (PPR) of $\widehat{\mathbf{A}}$,
  (14) \begin{align*}& \begin{split} \text{PPR}=\frac{\sum_{i\neq j}{\mathbf{I}_{\{\hat{a}_{ij} = 1 \& a_{ij} = 1\}}}}{\sum_{i\neq j}{\mathbf{I}_{\{\hat{a}_{ij} = 1\}}}}.\qquad\qquad\qquad \end{split}\end{align*}

- True Negative Rate (TNR) of $\widehat{\mathbf{A}}$,
  (15) \begin{align*}& \begin{split} \text{TNR}=\frac{\sum_{i\neq j}{\mathbf{I}_{\{\hat{a}_{ij} = -1 \& a_{ij} = -1\}}}}{\sum_{i\neq j}{\mathbf{I}_{\{a_{ij} = -1\}}}}.\qquad\qquad\qquad \end{split}\end{align*}

- Precise Negative Rate (PNR) of $\widehat{\mathbf{A}}$,
  (16) \begin{align*}& \begin{split} \text{PNR}=\frac{\sum_{i\neq j}{\mathbf{I}_{\{\hat{a}_{ij} = -1 \& a_{ij} = -1\}}}}{\sum_{i\neq j}{\mathbf{I}_{\{\hat{a}_{ij} = -1\}}}}.\qquad\qquad\qquad \end{split}\end{align*}

- Accuracy (ACC) of $\widehat{\boldsymbol{\Delta }}$,
  (17) \begin{align*}& \begin{split} \text{ACC}=\frac{\sum_{i\neq j}{\mathbf{I}_{\{\hat{\delta}_{ij} = \delta_{ij} \& \hat{a}_{ij} \neq 0\}}}}{\sum_{i\neq j}{\mathbf{I}_{\{\hat{a}_{ij} \neq 0\}}}}.\qquad\qquad\qquad \end{split}\end{align*}

Table 2 shows the performance of BBTD on five synthesized subtrees. Obviously, the pre-screening process can effectively screen around $70\%$ of $a_{ij}$ for each subtree. Also, $\text{FNR}=0$ in all settings indicates that there is no loss of ground truth regulatory relationships after implementing the pre-screening process. This robustness is attributable to the utilization of Fisher’s exact test, a nonparametric hypothesis testing method, which is inherently robust against variations in data distribution. Moreover, despite the variability in data distributions, the threshold value $\theta $ within the pre-screening process can be consistently set without losing any ground truth regulatory relationships. This consistency in performance demonstrates that the pre-screening process is robust across different data distributions. Furthermore, the fourth to the eighth columns in Table 2 reveal only minor differences across different data distributions when the probabilistic Boolean network model is applied, in conjunction with the use of the MCMC algorithm to infer unknown parameters. Table 2 shows that most gene regulations (positive and negative) as well as time delay can be predicted accurately by the MCMC algorithm for distinct subtrees. In conclusion, our simulation results demonstrate that the BBTD method exhibits robustness across these data variations.

**Table 2:** The simulation results for synthesized cell lineage subtrees; the second to the eighth columns summarize the denoted indices in Equation (11) to (17).

Subtree	PR	FNR	TPR	PPR	TNR	PNR	ACC
AB	0.683	0	0.970	0.837	0.976	0.759	0.894
C	0.699	0	0.976	0.853	0.970	0.764	0.887
D	0.705	0	0.969	0.953	0.949	0.865	0.969
E	0.732	0	0.965	0.918	0.954	0.843	0.942
MS	0.696	0	0.973	0.823	0.970	0.724	0.878

**Figure 4:**
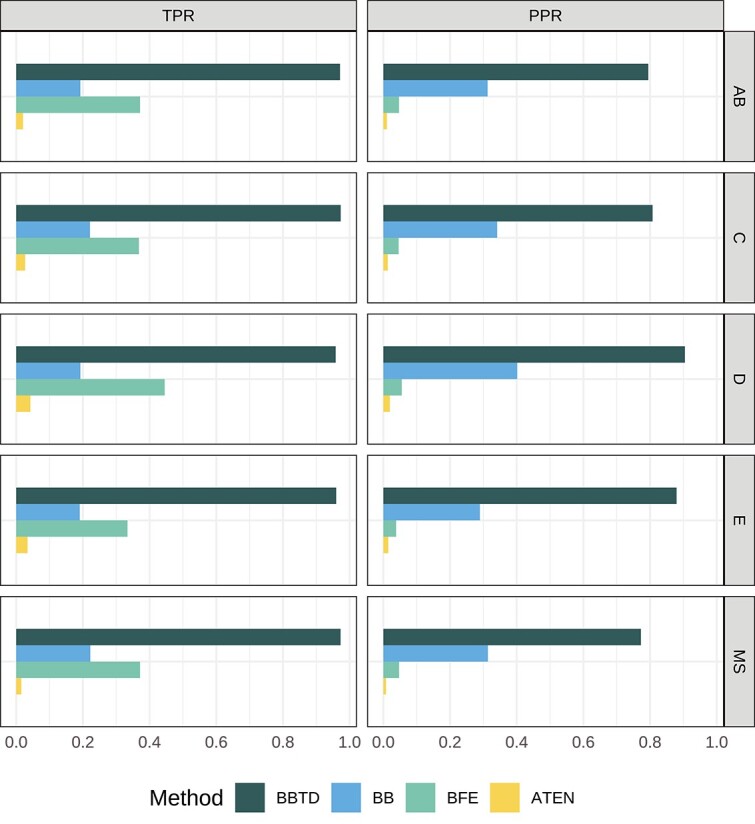
Performance of synthesized data with difference methods: BBTD, BB, BFE, and ATEN; the figure shows the comparisons of average TPR and average PPR for five synthesized subtrees.

In addition, the statistical model in BBTD is replaced with the probabilistic Boolean network model proposed by [17] for comparison which does not consider multi-time delay and this method is called BB. BBTD is also compared with other existing Boolean network inference algorithms mentioned in Section 2, i.e. REV, BFE, and ATEN. The details of algorithm settings are shown in Supplementary II. Since REV, BFE, and ATEN cannot distinguish positive and negative regulation relationships, for ease of comparison, we set $\hat{a}_{ij}=1$ if there is a regulation acting from regulator gene $j$ to regulated gene $i$, otherwise $\hat{a}_{ij}=0$. All experiments in this study are conducted on a server equipped with two CPUs (Inter (R) Xeon(R) Platinum 8368 with two threads * 38 cores, @ 2.40 GHz). The network inference results are displayed in Fig. 4. REV is not listed in this figure due to its poor capability to handle nondeterministic network models. Although BFE and ATEN have a better tolerance of noisy data compared with REV, they consider neither the parent–child cell relationships within the tree-shaped dataset nor the time delay of regulation, thus resulting in lower correct rates. Figure 4 shows that our method outperforms other algorithms in five synthesized subtrees. The information of calculation time and memory usage for our method, BBTD, alongside three other Boolean network methods: BB, BFE, and ATEN, is shown in Supplementary Table S3.

### Real data analysis

**Figure 5:**
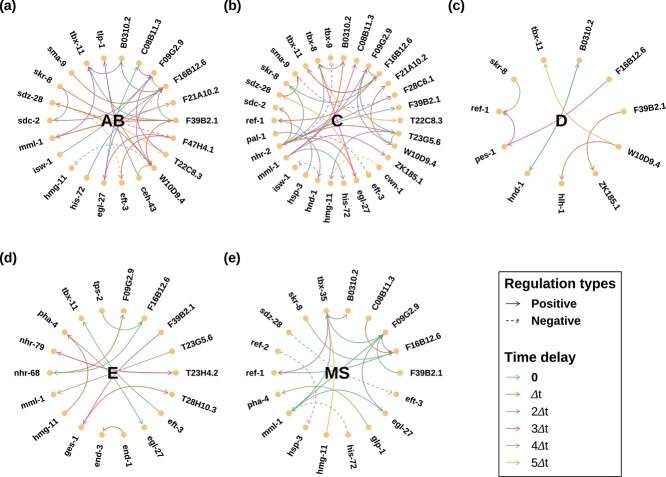
The inferred GRNs of the five cell lineage subtrees: ‘AB’, ‘C’, ‘D’, ‘E’, and ‘MS’; sub-figures (a) to (e) illustrate the GRNs of these subtrees in sequential order; each solid circle in the inferred GRN represents a gene; the presence of a solid (dashed) arrow indicates a positive (negative) regulatory relationship extending from regulator genes to regulated ones; the color of the arrow corresponds to the respective time delay associated with the regulatory relationship.

In this section, BBTD is further conducted to reconstruct GRNs for five cell lineage subtrees of *C. elegans* based on the 4D confocal microscopy data. The MCMC algorithm is implemented with five independent MCMC chains for each subtree. The trace plot of the log-posterior probabilities for these five MCMC chains is displayed in Supplementary Figure S3. The inferred results are displayed in Fig. 5. Each sub-figure showcases the directed positive or negative regulatory relationships, along with their corresponding time delay, from regulator genes to regulated genes. Specifically, there are 31 pairs gene regulatory relationships in the ‘AB’ subtree, 34 pairs in the ‘C’ subtree, 8 pairs in the ‘D’ subtree, 12 pairs in the ‘E’ subtree, and 18 pairs in the ‘MS’ subtree.

#### Identification of direct gene regulatory relationships

Our network structures reveal direct gene regulatory relationships within distinct subtrees, confirming several pairs of relationships established in prior genetic studies. Notably, in the ‘AB’ subtree, *sma-9* activates *F16B12.6*, a relationship validated in [42]. Similarly, in the ‘MS’ subtree, *glp-1* activates *pha-4*, as confirmed in [43]. Additionally, we identify that *egl-27* directly activates *tbx-8* in the ‘C’ subtree. This discovery is supported by the established interactions from *egl-27* to *tbx-9* and from *tbx-9* to *tbx-8*, both documented in [42]. Moreover, 34 pairs of regulatory relationships across five subtrees whose regulator and regulated genes have same function annotations are detected using gene ontology enrichment analysis [44] (see Supplementary Table S4 and Table S5). These findings provide support for the gene regulatory relationships identified in our analysis.

In order to compare our method with other available methods on real data, confirmed gene regulatory relationships among the candidate genes of each subtree are retrieved from BioGRID Version 4.4.233 [45] and WormBase Version WS254 [46], which are displayed in Supplementary Table S6. According to the results depicted in Table 3, our method, BBTD, successfully detects confirmed gene regulatory relationships in four subtrees, outperforming the other methods: BFE identifies confirmed relationships in three subtrees; BB detects them in two subtrees; and ATEN does not discover any confirmed relationships. For the ‘E’ subtree, although BBTD identifies only one regulatory relationship, resulting in a lower TPR than that achieved by BFE, it exhibits a significantly higher PPR. This distinction suggests that BFE, with its tendency to overpredict, adopts a greedy approach that leads to the identification of an excessive number of regulatory relationships within each subtree, potentially compromising precision.

**Table 3:** Comparison of gene regulatory relationships for five subtrees detected by BBTD and other three Boolean network inference methods: BB, BFE, and ATEN; the column labeled ‘Ground truth’ represents the number of confirmed gene regulatory relationships; each cell from the second to the fifth column contains three rows; the first row shows the number of predicted gene regulatory relationships; the value in parentheses indicates the overlap between the predicted relationships and the confirmed ones; the values in square brackets represent TPR and PPR, respectively, separated by a slash; the cells which are bold means there is at least one confirmed gene regulatory relationship.

Subtree	BBTD	BB	BFE	ATEN	Ground truth
AB	**31 (1) [0.333/0.032]**	**28 (1) [0.333/0.036]**	142 (0) [0/0]	29 (0) [0/0]	3
C	**34 (1) [0.167/0.029]**	19 (0) [0/0]	**171 (1) [0.167/0.006]**	41 (0) [0/0]	6
D	8 (0) [0/0]	7 (0) [0/0]	**117 (1) [0.25/0.009]**	23 (0) [0/0]	4
E	**12 (1) [0.111/0.083]**	**12 (1) [0.111/0.083]**	**290 (4) [0.444/0.014]**	64 (0) [0/0]	9
MS	**18 (1) [0.167/0.056]**	19 (0) [0/0]	164 (0) [0/0]	33 (0) [0/0]	6

#### Heterogeneity of regulatory relationships in different cell lineage subtrees

Based on our findings, it is evident that diverse regulatory relationships characterize different subtrees, a diversity likely attributed to inherent heterogeneity among them. On the one hand, the genes involved in regulation vary across distinct subtrees, reflecting their association with specific cell fates. In essence, a regulated gene within a given subtree may engage distinct regulator genes for its regulation compared with other subtrees. Consider *ref-1* as an illustrative example: in the ‘C’ subtree, it is activated by *hsp-3*, expressed in various structures including hypodermis, while activation in the ‘D’ subtree is mediated by *pes-1*, expressed in muscle. This variance may be linked to the differing cell fates of the subtrees. On the other hand, it is apparent that regulatory relationships involving the same regulator-regulated genes can manifest variations across different subtrees. For instance, although *F09G2.9* positively activates *F16B12.6* in both ‘C’ and ‘MS’ subtrees, the time delay is zero in the ‘C’ subtree, in contrast to two units in the ‘MS’ subtree. This discrepancy implies that the activation of *F16B12.6* by *F09G2.9* takes more time in the ‘MS’ subtree than in the ‘C’ subtree.

#### Potentially significant gene relationships associated with human diseases

In recent years, scientists have gained valuable insights into human disease genes through the study of *C. elegans* [47, 48]. This research discovers some regulatory relationships among genes whose human orthologs play a crucial role in human disease pathogenesis. For instance, *hmg-11* corresponds to the human gene HMGA2, implicated in various diseases, including lipoma [49], leiomyoma [50], and ovarian cancer [51]. As depicted in Fig. 5a and b, *hmg-11* is suppressed by *W10D9.4* in the ‘AB’ subtree and activated by *T23G5.6* with a two-unit time delay in the ‘C’ subtree. Consequently, potential interventions such as inducing the expression of the human ortholog of *W10D9.4* or mitigating the regulatory effect of the human ortholog of *T23G5.6* within the specified time frame could be considered to modulate the expression of HMGA2 in the treatment of related diseases in the future.

## Discussion

In this study, a novel data integration framework is presented to effectively align the gene expression data for each cell lineage subtree, addressing the challenge of non-pairwise time series across various data files. Then, a new method BBTD is proposed to reconstruct GRNs from pairwise binarized data by establishing a probabilistic Boolean network model with time delay. Through extensive simulation studies, the performance of our method is demonstrated across varying the number of genes and cells, which outperforms other Boolean network inference methods. When applied to the real dataset, our method successfully identifies known regulatory relationships between regulator genes and regulated genes. Besides, the results of spatial-temporal regulation reveal the heterogeneity of regulatory relationships in different cell subtrees, providing critical insights into cellular functions and differentiation processes. Moreover, the identification of disease-related gene regulatory relationships provides opportunities for the development of novel diagnostics and precision medicine.

To address the issue of non-pairwise time series data, a framework of data integration including interpolation and binarization is proposed. Interpolation helps preserve more information; however, the absence of pairwise data points necessitates extensive interpolations, which can introduce considerable noise. This noise can severely compromise the effectiveness of more detailed analyses, such as applying Bayesian networks to the interpolated fluorescence intensities. Consequently, binarizing the data after interpolation and then employing Boolean networks presents a robust strategy for dealing with such high-resolution but inherently noisy datasets typical in this study. Moreover, our model excels in performance as it not only accounts for the influence of noise, enhancing its reliability, but also incorporates time delay as a discrete parameter, which closely reflects the dynamics of real biological systems. Furthermore, BBTD can infer the positive and negative regulation, while many other existing methods only determine the presence of a regulatory relationship between two genes.

However, our study is subject to several limitations. Due to the application of data integration, discretization of the gene expression state inevitably results in some degree of information loss, which hinders us from further investigating GRNs in more details. In addition, as binarization does not capture the continuous and often subtle variations in gene expression levels, which are crucial for detecting feedback mechanisms, self-regulation of genes is not considered in our study. This limitation restricts the model’s ability to fully capture the complexities of real biological systems. Last but not least, since a great number of parameters in the proposed model are discrete (e.g. $\mathbf{A}=\left (a_{ij}\right )$), it is difficult to judge the convergence of MCMC chains in a more principled way. In further work, more specific gene regulatory models, which can also handle non-pairwise datasets, need to be proposed to delve deeper into the mechanisms of gene regulation.

**Key Points**

- The research addresses the challenge of inferring GRNs using tree-shaped gene expression data from time-lapse confocal laser microscopy of *C. elegans*, offering a solution to handle such high-resolution but rather noisy data.
- A comprehensive framework for data integration as well as a novel Bayesian approach based on Boolean network with time delay are proposed, enhancing the accuracy of GRNs construction.
- Our method outperforms existing Boolean network algorithms in both simulation studies and real data analysis, validating known gene regulatory relationships and uncovering the heterogeneity of regulatory relationships across different subtrees.
- All source code is freely available at the GitHub repository https://github.com/edawu11/BBTD.git for promoting reproducibility.

## Supplementary Material

supplementary_bbae396

## Data Availability

The datasets are available via http://epic.gs.washington.edu/. The processed data is available via https://doi.org/10.5281/zenodo.11261241. All source code is available at https://github.com/edawu11/BBTD.git.
