# Supplementary material for: Reconstruction of gene regulatory networks for Caenorhabditis elegans using tree-shaped gene expression data
Source: Brief Bioinform. 2024 Aug 12;25(5):bbae396. doi: 10.1093/bib/bbae396 (PMC11318059; doi:10.1093/bib/bbae396)
Supplement: supplementary_bbae396 [file supplementary_bbae396.pdf]

Supplemental Material
Reconstruction of gene regulatory networks for *Caenorhabditis elegans* using tree-shaped gene expression data

Yida Wu[1], Da Zhou[1] and Jie Hu[1,*]
[1]School of Mathematical Sciences, Xiamen University, Xiamen 361005, China
*To whom correspondence should be addressed.

I Identification of candidate cells and genes

Upon integrating the raw tree-shaped data for each subtree, it is observed that certain genes fail to express in specific cells, resulting in what we define as missing values. Figure S1 depicts the missing values of each gene in every cell for five real subtree datasets. To ensure that the proportion of missing values within each subtree is less than 5%, several cells and genes are removed. First, cells with more than 55% of missing genes are excluded in each subtree. The retained cells form the pool of candidate cells for analysis, as presented in Table S1. Notably, Table S1 showcases a subset of candidate cells characterized by distinct cell fates. Subsequently, based on the candidate cells, genes missing in more than 20% of cells are removed, and the remaining genes constitute the pool of candidate genes, as detailed in Table S2. Figure S2 illustrates the distribution of missing values for each candidate gene across all candidate cells in five subtrees.

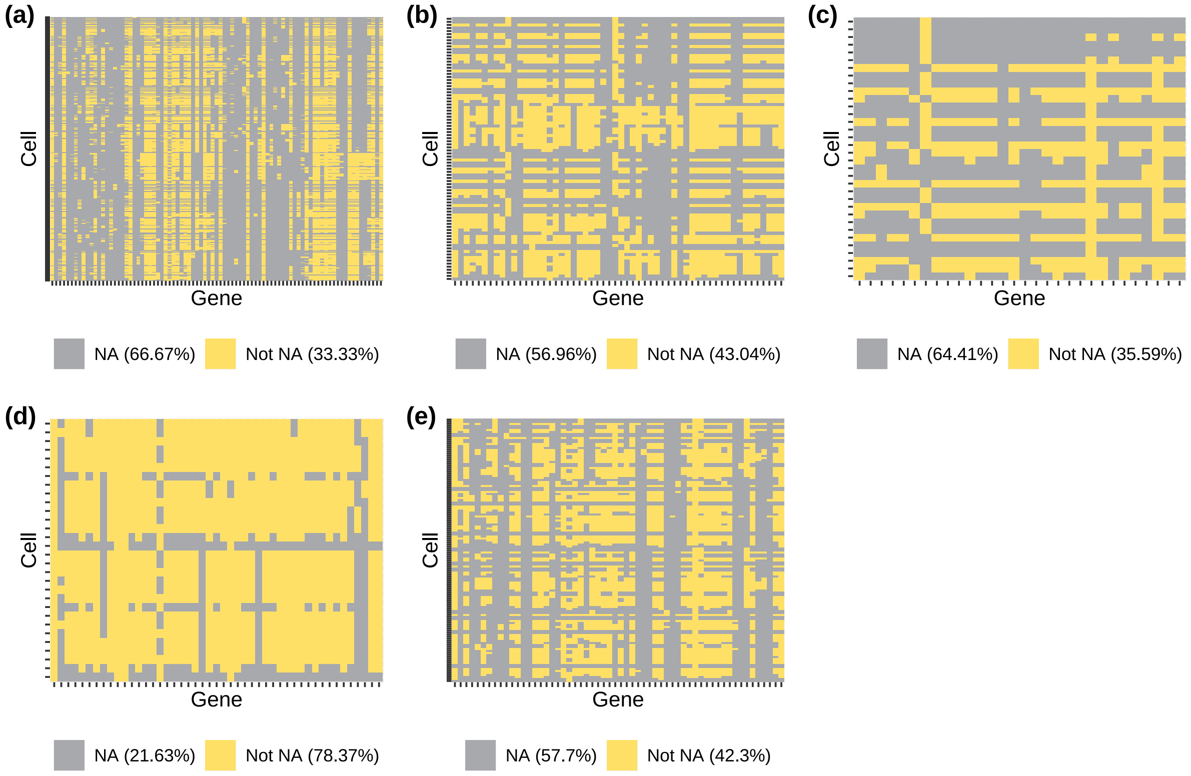

Figure S1: Missing value plots for five real subtree datasets. The X-axis represents gene and the Y-axis represents cell. Gene and cell names are omitted in each sub-figure. 'NA' indicates that the gene is missing in the cell, while 'Not NA' means that the gene has a time series of expression rates in the cell. The percentages in parentheses represent the ratio of 'NA' or 'Not NA'. (a) Missing value plot of the 'AB' subtree. (b) Missing value plot of the 'C' subtree. (c) Missing value plot of the 'D' subtree. (d) Missing value plot of the 'E' subtree. (e) Missing value plot of the 'MS' subtree.

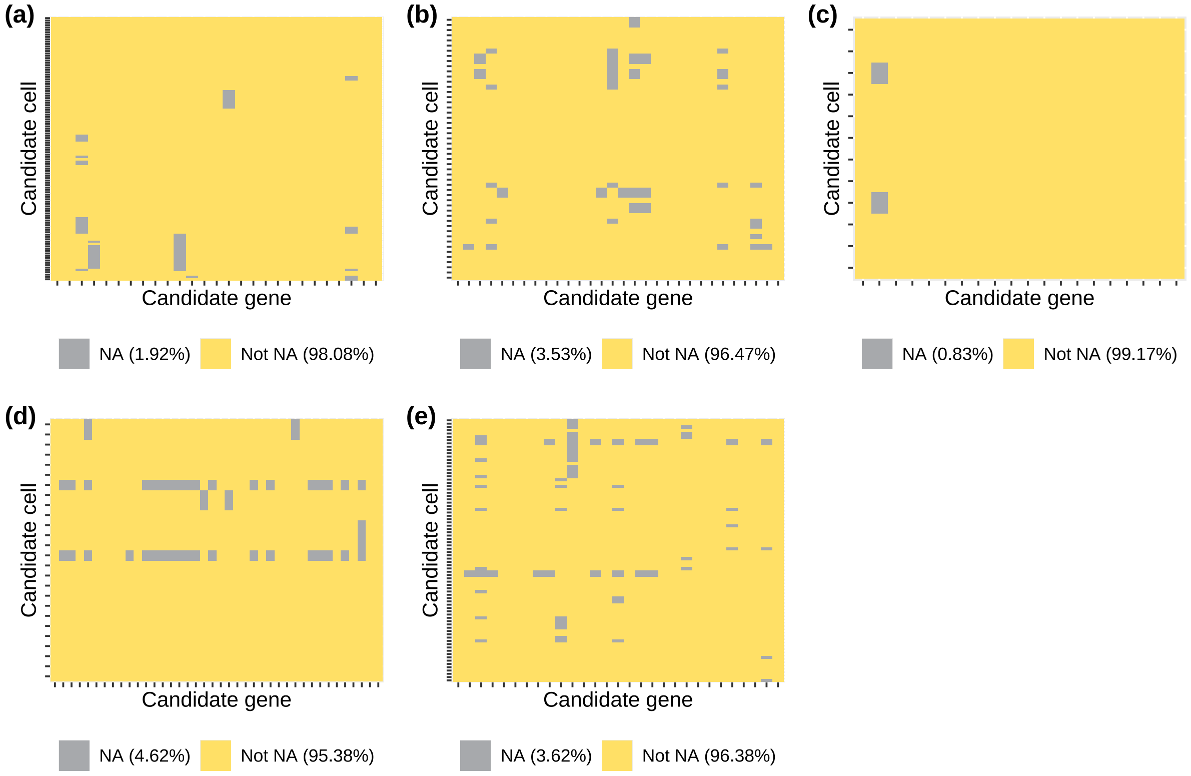

Figure S2: Missing value plots for five real subtree datasets after identifying the candidate cells and genes. The X-axis represents candidate gene and the Y-axis represents candidate cell. Gene and cell names are omitted in each sub-figure. 'NA' indicates that the gene is missing in the cell, while 'Not NA' means that the gene has a time series of expression rates in the cell. The percentages in parentheses represent the ratio of 'NA' or 'Not NA'. (a) Missing value plot of the 'AB' subtree. (b) Missing value plot of the 'C' subtree. (c) Missing value plot of the 'D' subtree. (d) Missing value plot of the 'E' subtree. (e) Missing value plot of the 'MS' subtree.

Table S1: Candidate cells with cell fates for five real subtree datasets.

Subtree	Candidate cell	Cell fate
AB	ABplapaapa, ABplapapap, ABplapappa, ABplappaaa, ABplappaap, ABprapaapp, ABprapapap, ABprapappa, ABprappaaa, ABprappaap, ABprappapp	Blast cells
	ABplaappaa, ABplaappap, ABpraappaa, ABpraappap, ABarpappaa, ABplaapppa, ABplaapppp, ABplappppa, ABpraapppa, ABpraapppp, ABprappppa, ABarppaaap, ABarpppaap, ABarppapaa, ABarppapap, ABarppappa, ABarppappp, ABarpppaa, ABarppppap, ABarpppppa, ABarpppppp, ABplappapa, ABprappapa, ABplappppp	Hypodermis
	ABarppaaaa, ABarpppaaa, ABprapaapa	Nerve tissue
C	Caapa	Death
	Caappv	Ganglion
	Caaaaa, Caaaap, Caaapa, Caaapp, Cpaaaa, Cpaaap, Cpaapa, Cpaapp, Cpapaa, Cpapap, Cpappd, Cpappv, Caappd	Hypodermis
	Cppaaa, Cppaap, Cppapa, Cppapp, Cpppaa, Cpppap, Cppppa, Cppppp, Capaaa, Capaap, Capapa, Capapp, Cappaa, Cappap, Capppa, Capppp	Muscle
D	Daaa, Daap, Dapa, Dapp, Dpaa, Dpap, Dppa, Dppp	Muscle
E	Ealaa, Ealap, Ealpa, Ealpp, Earaa, Earap, Earpa, Earpp, Eplaa, Eplap, Eplpa, Eplpp, Epraa, Eprap, Eprpa, Eprpp	Intestine
MS	MSppapaa, MSapapaa	Coelomocytes
	MSpppaa	Death
	MSppaaaa, MSaaaaaa, MSapaaaa	Ganglion
	MSappaa	Mesoderm
	MSppppp, MSppappa, MSppappp, MSppapap, MSppaaap, MSpappa, MSapppp, MSappap, MSapappp, MSapappa, MSapapap, MSapaaap, MSaappp, MSpppap	Muscle
	MSpapapp, MSpapaaa, MSpaapaa, MSpaapap, MSpaaapa, MSpaaapp, MSpaaaaa, MSpaaaap, MSaaaaap, MSaaaapa, MSaaaapp, MSaaapaa, MSaaapap, MSaaapp, MSaapaaa, MSaapaap, MSaapapa, MSaapapp, MSaappa, MSpapaap, MSpapapa	Pharynx

Table S2: Candidate genes used for gene regulatory network inference for each subtree.

Subtree	Candidate gene
AB	*B0310.2, C08B11.3, ceh-43, eft-3, egl-27, F09G2.9, F16B12.6, F21A10.2, F28C6.1, F39B2.1, F47H4.1, glp-1, his-72, hmg-11, hsp-3, isw-1, mml-1, nhr-2, sdc-2, sdz-28, skr-8, sma-9, T22C8.3, tbx-11, tlp-1, W10D9.4, ZK185.1*
C	*B0310.2, C08B11.3, cwn-1, eft-3, egl-27, F09G2.9, F16B12.6, F21A10.2, F28C6.1, F39B2.1, his-72, hmg-11, hnd-1, hsp-3, isw-1, mml-1, nhr-2, pal-1, ref-1, sdc-2, sdz-28, skr-8, sma-9, T22C8.3, T23G5.6, tbx-11, tbx-8, tbx-9, W10D9.4, ZK185.1*
D	*B0310.2, C08B11.3, F09G2.9, F16B12.6, F21A10.2, F28C6.1, F39B2.1, hlh-1, hnd-1, mml-1, nhr-2, pal-1, pes-1, ref-1, sdc-2, skr-8, sma-9, tbx-11, W10D9.4, ZK185.1*
E	*B0310.2, C08B11.3, dpy-31, dve-1, eft-3, egl-27, elt-7, end-1, end-3, F09G2.9, F16B12.6, F17C11.1, F21A10.2, F39B2.1, ges-1, glp-1, his-72, hmg-11, hsp-3, mel-28, mml-1, nhr-2, nhr-57, nhr-68, nhr-79, pgp-2, pha-4, ref-1, sdc-2, sdz-28, skr-8, sma-9, T22C8.3, T23G5.6, T23H4.2, T28H10.3, tbx-11, tbx-8, tps-2, ZK185.1*
MS	*B0310.2, C08B11.3, eft-3, egl-27, F09G2.9, F16B12.6, F21A10.2, F28C6.1, F39B2.1, F47H4.1, glp-1, his-72, hlh-1, hmg-11, hsp-3, isw-1, mml-1, nhr-2, pha-4, ref-1, ref-2, sdc-2, sdz-28, skr-8, sma-9, T22C8.3, tbx-35, W10D9.4, ZK185.1*

II Algorithm settings

To validate BBTD, four Boolean network methods are used to compare the performance with BBTD, including BB [1], REV [2], BFE [3] and ATEN [4]. The model of BB is given as follows:
If $H_i^{t_k^r} = 0$,

$$\mathrm{P}\left(s_i^{t_k^r} = s_i^{t_k - \Delta t} \mid s_i^{t_k^r - \Delta t}\right) = \frac{1}{1 + e^{-\alpha}}; \tag{S2}$$

and if $H_i^{t_k^r} \neq 0$,

$$\mathrm{P}\left(s_i^{t_k^r} \mid s_1^{t_k^r - \Delta t}, \ldots, s_N^{t_k^r - \Delta t}\right) = \frac{\exp\left[\beta(2s_i^{t_k^r} - 1)H_i^{t_k^r}\right]}{\exp(\beta H_i^{t_k^r}) + \exp(-\beta H_i^{t_k^r})}. \tag{S3}$$

where $s_i^{t_k^r} \in \{0, 1\}$ denotes the expression state of gene $i(i = 1, \ldots, N)$ at time $t_k^r(r = 1, \ldots, R, k = 2, \ldots, K^r)$. All unknown parameters are estimated by our proposed prescreening process and the Bayesian inference framework. Both REV and BFE are implemented in the R package BoolNet [5]. Since the poor capability to handle nondeterministic network models, REV is not used in all datasets. ATEN is run using the R package ATEN. The default parameters are used for most of the software packages. For example, the parameter maxK of BFE is set to 5, and the number of iterations of ATEN is set to 5,000.

III Comparation of four Boolean network inference methods

In this study, BBTD, BB, BFE and ATEN are applied to the synthesized datatsets of *C. elegans*. Table S3 summarizes the information of calculation time and memory usage these four methods.

Table S3: Average calculation time and memory usage for BBTD and three other Boolean network inference methods across five synthesized subtree datasets. The value in parentheses indicates the memory usage.

Subtree	BBTD	BB	BFE	ATEN
AB	42 h (2.30 GB)	3 h (1.19 GB)	2 s (0.55 GB)	47 h (1.43 GB)
C	32 h (1.46 GB)	2 h (1.01 GB)	3 s (0.54 GB)	20 h (1.36 GB)
D	11 h (1.38 GB)	1 h (1.00 GB)	< 1 s (0.54 GB)	5 h (1.34 GB)
E	43 h (1.45 GB)	3 h (1.00 GB)	24 s (0.47 GB)	11 h (1.35 GB)
MS	39 h (1.23 GB)	2 h (1.15 GB)	2 s (0.53 GB)	33 h (1.38 GB)

IV Trace plots of the log-posterior probability of five MCMC chains for five real subtree datasets

Figure S3 shows the log-posterior probability of five MCMC chains for each real subtree datasets. It is observed that the chains converged after about 10,000 iterations.

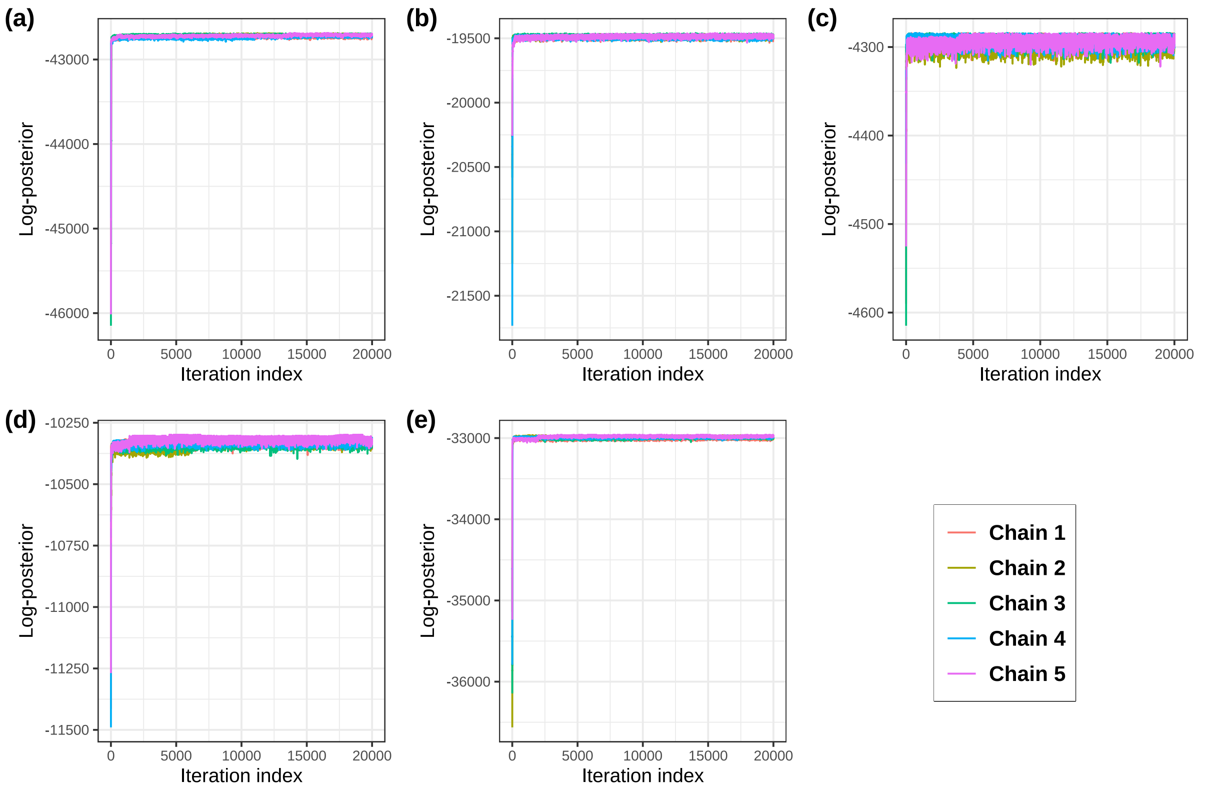

Figure S3: Trace plots of the log-posterior probability of five MCMC chains for five real subtree datasets. Each chain is run for 20,000 iterations. (a) Trace plots of the 'AB' subtree. (b) Trace plots of the 'C' subtree. (c) Trace plots of the 'D' subtree. (d) Trace plots of the 'E' subtree. (e) Trace plots of the 'MS' subtree.

V Gene ontology enrichment analysis

In this study, gene ontology (GO) enrichment analysis is conducted using clusterProfiler v4.6.2 [6]. The cutoffs for the p-value and q value are set at 0.05 and 0.20, respectively. The complete results of significant over-representation of functional categories related to each pair of regulator-regulated genes are presented in Table S4. The corresponding descriptions of GO ID within Table S4 are provided in Table S5.

Table S4: 34 pairs of regulatory relationships across five subtrees whose regulator and regulated genes have same function annotations.

Subtree	Regulator gene	Regulated gene	GO ID
AB	*B0310.2*	*tbx-11*	GO:0000978, GO:0000987, GO:0000977, GO:0000976, GO:0001067, GO:1990837, GO:0003690
AB	*F39B2.1*	*W10D9.4*	GO:0000978, GO:0000987, GO:0000981, GO:0000977, GO:0000976, GO:0001067, GO:1990837, GO:0003690
AB	*F39B2.1*	*mml-1*	GO:0000978, GO:0000987, GO:0000981, GO:0000977, GO:0000976, GO:0001067, GO:1990837, GO:0003690
AB	*F39B2.1*	*tbx-11*	GO:0000978, GO:0000987, GO:0000981, GO:0000977, GO:0000976, GO:0001067, GO:1990837, GO:0003690
AB	*ceh-43*	*W10D9.4*	GO:0000978, GO:0000987, GO:0000981, GO:0000977, GO:0000976, GO:0001067, GO:1990837, GO:0003690
AB	*egl-27*	*C08B11.3*	GO:0070603, GO:1904949, GO:0000785, GO:0005694
AB	*mml-1*	*tbx-11*	GO:0000978, GO:0000987, GO:0000981, GO:0000977, GO:0000976, GO:0001067, GO:1990837, GO:0003690
AB	*tlp-1*	*W10D9.4*	GO:0045892, GO:1902679, GO:1903507, GO:0051253, GO:0045934, GO:0010558, GO:0045944, GO:0031327, GO:0009890, GO:0045893, GO:1902680, GO:1903508, GO:0010557, GO:0009891, GO:0031328, GO:0051254, GO:0031324, GO:0045935, GO:0051172, GO:0051173
C	*B0310.2*	*tbx-8*	GO:0000978, GO:0000987, GO:0000977, GO:0000976, GO:0001067, GO:1990837, GO:0003690
C	*W10D9.4*	*F21A10.2*	GO:0045893, GO:1902680, GO:1903508, GO:0010557, GO:0009891, GO:0031328, GO:0051254, GO:0045935, GO:0051173
C	*egl-27*	*tbx-8*	GO:0010171, GO:0001708, GO:0045165, GO:0005667
C	*hnd-1*	*isw-1*	GO:0051094, GO:0048513
C	*mml-1*	*B0310.2*	GO:0000978, GO:0000987, GO:0000977, GO:0000976, GO:0001067, GO:1990837, GO:0003690
C	*nhr-2*	*F28C6.1*	GO:0045944, GO:0045893, GO:1902680, GO:1903508, GO:0010557, GO:0009891, GO:0031328, GO:0051254, GO:0045935, GO:0051173, GO:0000981, GO:0000977, GO:0000976, GO:0001067, GO:1990837, GO:0003690
C	*nhr-2*	*F39B2.1*	GO:0000978, GO:0000987, GO:0000981, GO:0000977, GO:0000976, GO:0001067, GO:1990837, GO:0003690
C	*nhr-2*	*egl-27*	GO:0045892, GO:1902679, GO:1903507, GO:0051253, GO:0045934, GO:0010558, GO:0031327, GO:0009890, GO:0031324, GO:0051172
C	*nhr-2*	*sdc-2*	GO:0000122, GO:0045892, GO:1902679, GO:1903507, GO:0051253, GO:0045934, GO:0010558, GO:0031327, GO:0009890, GO:0031324, GO:0051172
C	*nhr-2*	*tbx-8*	GO:0000978, GO:0000987, GO:0000981, GO:0000977, GO:0000976, GO:0001067, GO:1990837, GO:0003690
C	*nhr-2*	*tbx-9*	GO:0000978, GO:0000987, GO:0000981, GO:0000977, GO:0000976, GO:0001067, GO:1990837, GO:0003690
C	*pal-1*	*hnd-1*	GO:0042692, GO:0061061, GO:0009888, GO:0048513, GO:0000981, GO:0000977, GO:0000976, GO:0001067, GO:1990837, GO:0003690
C	*sdz-28*	*sma-9*	GO:0007369, GO:0048598
C	*sma-9*	*F28C6.1*	GO:0045893, GO:1902680, GO:1903508, GO:0010557, GO:0009891, GO:0031328, GO:0051254, GO:0045935, GO:0051173
D	*B0310.2*	*hnd-1*	GO:0000977, GO:0000976, GO:0001067, GO:1990837, GO:0003690
D	*W10D9.4*	*hlh-1*	GO:0009888, GO:0045944, GO:0045893, GO:1902680, GO:1903508, GO:0010557, GO:0009891, GO:0031328, GO:0051254, GO:0045935, GO:0051173, GO:0046983, GO:0000978, GO:0000987, GO:0000981, GO:0000977, GO:0000976, GO:0001067, GO:1990837, GO:0003690
D	*W10D9.4*	*tbx-11*	GO:0000978, GO:0000987, GO:0000981, GO:0000977, GO:0000976, GO:0001067, GO:1990837, GO:0003690
D	*hnd-1*	*B0310.2*	GO:0000977, GO:0000976, GO:0001067, GO:1990837, GO:0003690
D	*pes-1*	*ref-1*	GO:0000978, GO:0000987, GO:0000981, GO:0000977, GO:0000976, GO:0001067, GO:1990837, GO:0003690
E	*end-1*	*end-3*	GO:0001714, GO:0001706, GO:0001711, GO:0035987, GO:0007492, GO:0060795, GO:0001704, GO:0007369, GO:0048646, GO:0001708, GO:0048598, GO:0009888, GO:0045944, GO:0045165, GO:0045893, GO:1902680, GO:1903508, GO:0010557, GO:0009891, GO:0031328, GO:0051254, GO:0045935, GO:0051173, GO:0000981
E	*pha-4*	*egl-27*	GO:0001708, GO:0045165
MS	*glp-1*	*pha-4*	GO:0060465, GO:0048565, GO:0055123, GO:0035295, GO:0007389, GO:0010628, GO:0001708, GO:0010608, GO:0045944, GO:0045165, GO:0045893, GO:1902680, GO:1903508, GO:0010557, GO:0009891, GO:0031328, GO:0051254, GO:0045935, GO:0051173
MS	*mml-1*	*egl-27*	GO:0045892, GO:1902679, GO:1903507, GO:0051253, GO:0045934, GO:0010558, GO:0031327, GO:0009890, GO:0031324, GO:0051172
MS	*mml-1*	*tbx-35*	GO:0000978, GO:0000987, GO:0000981, GO:0000977, GO:0000976, GO:0001067, GO:1990837, GO:0003690
MS	*tbx-35*	*B0310.2*	GO:0000978, GO:0000987, GO:0000977, GO:0000976, GO:0001067, GO:1990837, GO:0003690
MS	*tbx-35*	*ref-1*	GO:0001708, GO:0009888, GO:0045165, GO:0000978, GO:0000987, GO:0000981, GO:0000977, GO:0000976, GO:0001067, GO:1990837, GO:0003690

Table S5: Description of GO ID.

GO ID	Description
GO:0000122	Negative regulation of transcription by RNA polymerase ii
GO:0000785	Chromatin
GO:0000976	Transcription cis-regulatory region binding
GO:0000977	RNA polymerase ii transcription regulatory region sequence-specific DNA binding
GO:0000978	RNA polymerase ii cis-regulatory region sequence-specific DNA binding
GO:0000981	DNA-binding transcription factor activity, RNA polymerase ii-specific
GO:0000987	Cis-regulatory region sequence-specific DNA binding
GO:0001067	Transcription regulatory region nucleic acid binding
GO:0001704	Formation of primary germ layer
GO:0001706	Endoderm formation
GO:0001708	Cell fate specification
GO:0001711	Endodermal cell fate commitment
GO:0001714	Endodermal cell fate specification
GO:0003690	Double-stranded DNA binding
GO:0005667	Transcription regulator complex
GO:0005694	Chromosome
GO:0007369	Gastrulation
GO:0007389	Pattern specification process
GO:0007492	Endoderm development
GO:0009888	Tissue development
GO:0009890	Negative regulation of biosynthetic process
GO:0009891	Positive regulation of biosynthetic process
GO:0010171	Body morphogenesis
GO:0010557	Positive regulation of macromolecule biosynthetic process
GO:0010558	Negative regulation of macromolecule biosynthetic process
GO:0010608	Post-transcriptional regulation of gene expression
GO:0010628	Positive regulation of gene expression
GO:0031324	Negative regulation of cellular metabolic process
GO:0031327	Negative regulation of cellular biosynthetic process
GO:0031328	Positive regulation of cellular biosynthetic process
GO:0035295	Tube development
GO:0035987	Endodermal cell differentiation
GO:0042692	Muscle cell differentiation
GO:0045165	Cell fate commitment
GO:0045892	Negative regulation of DNA-templated transcription
GO:0045893	Positive regulation of DNA-templated transcription
GO:0045934	Negative regulation of nucleobase-containing compound metabolic process
GO:0045935	Positive regulation of nucleobase-containing compound metabolic process
GO:0045944	Positive regulation of transcription by RNA polymerase ii
GO:0046983	Protein dimerization activity
GO:0048513	Animal organ development
GO:0048565	Digestive tract development
GO:0048598	Embryonic morphogenesis
GO:0048646	Anatomical structure formation involved in morphogenesis
GO:0051094	Positive regulation of developmental process
GO:0051172	Negative regulation of nitrogen compound metabolic process
GO:0051173	Positive regulation of nitrogen compound metabolic process
GO:0051253	Negative regulation of RNA metabolic process
GO:0051254	Positive regulation of RNA metabolic process
GO:0055123	Digestive system development
GO:0060465	Pharynx development
GO:0060795	Cell fate commitment involved in formation of primary germ layer
GO:0061061	Muscle structure development
GO:0070603	Swi/snf superfamily-type complex
GO:1902679	Negative regulation of RNA biosynthetic process
GO:1902680	Positive regulation of RNA biosynthetic process
GO:1903507	Negative regulation of nucleic acid-templated transcription
GO:1903508	Positive regulation of nucleic acid-templated transcription
GO:1904949	Atpase complex
GO:1990837	Sequence-specific double-stranded DNA binding

VI Confirmed gene regulatory relationships

Table S6: Confirmed gene regulatory relationships among the candidate genes of each subtree retrieved from BioGRID Version 4.4.233 and WormBase Version WS254.

Subtree	Regulator gene	Regulated gene	Database
AB	*glp-1*	*egl-27*	BioGRID
AB	*sma-9*	*F16B12.6*	BioGRID
AB	*sma-9*	*ceh-43*	BioGRID
C	*sma-9*	*F16B12.6*	BioGRID
C	*tbx-9*	*tbx-8*	BioGRID
C	*T22C8.3*	*tbx-9*	BioGRID
C	*egl-27*	*tbx-9*	BioGRID
C	*tbx-8*	*tbx-9*	WormBase
C	*pal-1*	*hnd-1*	WormBase
D	*sma-9*	*F16B12.6*	BioGRID
D	*hnd-1*	*hlh-1*	WormBase
D	*pal-1*	*hlh-1*	WormBase
D	*pal-1*	*hnd-1*	WormBase
E	*glp-1*	*egl-27*	BioGRID
E	*glp-1*	*pha-4*	BioGRID
E	*sma-9*	*F16B12.6*	BioGRID
E	*pha-4*	*F16B12.6*	BioGRID
E	*pha-4*	*tbx-8*	BioGRID
E	*end-1*	*end-3*	WormBase
E	*end-3*	*end-1*	WormBase
E	*ref-1*	*glp-1*	WormBase
E	*pha-4*	*glp-1*	WormBase
MS	*glp-1*	*egl-27*	BioGRID
MS	*glp-1*	*pha-4*	BioGRID
MS	*sma-9*	*F16B12.6*	BioGRID
MS	*pha-4*	*F16B12.6*	BioGRID
MS	*ref-1*	*glp-1*	WormBase
MS	*pha-4*	*glp-1*	WormBase

References

[1] Zhang Y, Qian M, Ouyang Q, *et al.* Stochastic model of yeast cell cycle network. *Physica D* 2006;**219**:35–39.

[2] Liang S, Fuhrman S, Somogyi R. Reveal, a general reverse engineering algorithm for inference of genetic network architectures. *Pac Symp Biocomput* 1998;**3**:18–29.

[3] Lähdesmäki H, Shmulevich I, Yli-Harja O. On learning gene regulatory networks under the Boolean network model. *Mach Learn* 2003;**52**:147–167.

[4] Shi N, Zhu Z, Tang K, *et al.* ATEN: and/or tree ensemble for inferring accurate Boolean network topology and dynamics. *Bioinformatics* 2020;**36**:578–585.

[5] Müssel C, Hopfensitz M, Kestler HA. BoolNet—an R package for generation, reconstruction and analysis of Boolean networks. *Bioinformatics* 2010;**26**:1378–1380.

[6] Wu T, Hu E, Xu S, *et al.* clusterProfiler 4.0: a universal enrichment tool for interpreting omics data. *Innovation* 2021;**2**:100141.
